# Supplementary material for: Dual PPRαϒ Agonists for the Management of Dyslipidemia: A Systematic Review and Meta-Analysis of Randomized Clinical Trials
Source: J Clin Med. 2023 Aug 31;12(17):5674. doi: 10.3390/jcm12175674 (PMC10488550; doi:10.3390/jcm12175674)

SUPPLEMENTARY MATERIAL

Journal of Clinical Medicine

Dual PPRαϒ Agonists for the Management of Dyslipidemia: A Systematic Review and Meta-Analysis of Randomized Clinical Trials

Antonio da Silva Menezes Junior [1,2,*,†], Vinícius Martins Rodrigues Oliveira [1,†], Izadora Caiado Oliveira [1], André Maroccolo de Sousa [1], Ana Júlia Prego Santana [1], Davi Peixoto Craveiro Carvalho [1], Ricardo Figueiredo Paro Piai [1], Fernando Henrique Matos [1], Arthur Marot de Paiva [1] and Gabriel Baêta Branquinho Reis [1]

[1] Faculty of Medicine, Federal University of Goiás, Goiânia 74605020, Brazil
[2] School of Medical and Life Sciences, Pontifical Catholic University of Goiás, Goiânia 74605050, Brazil
* Correspondence: a.menezes.junior@uol.com.br; Tel.: +55-62982711177
† These authors contributed equally to this work.

Table of Contents

Figure S1.. ***5***
Figure S2.. ***7***
Figure S3.. ***8***
Figure S4.. ***6***
Figure S5.. ***8***

Figure S1.

Funnel plots comparing the (A) triglyceride, (B) low-density lipoprotein cholesterol, (C) high-density lipoprotein cholesterol, and (D) fasting plasma glucose levels between the 2 mg and 4 mg saroglitazar groups. CI: Confidence interval; MD: Mean difference; SE: Standard error.

A

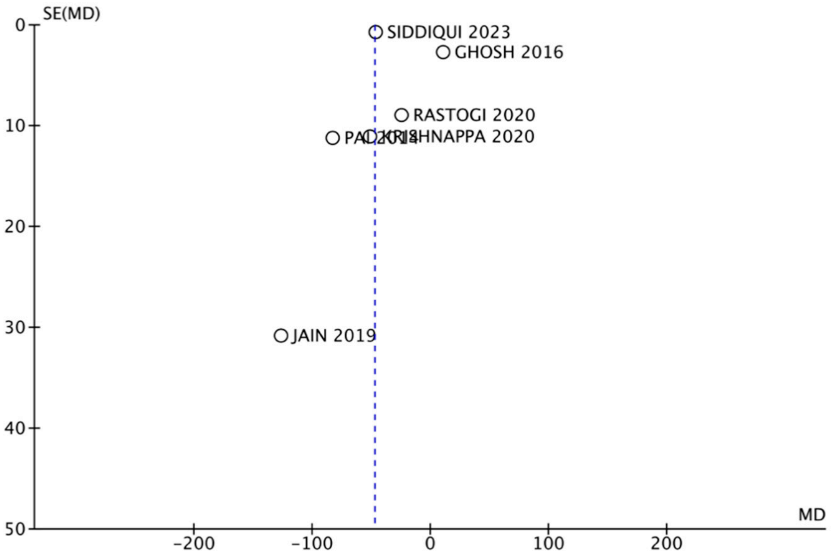

B.

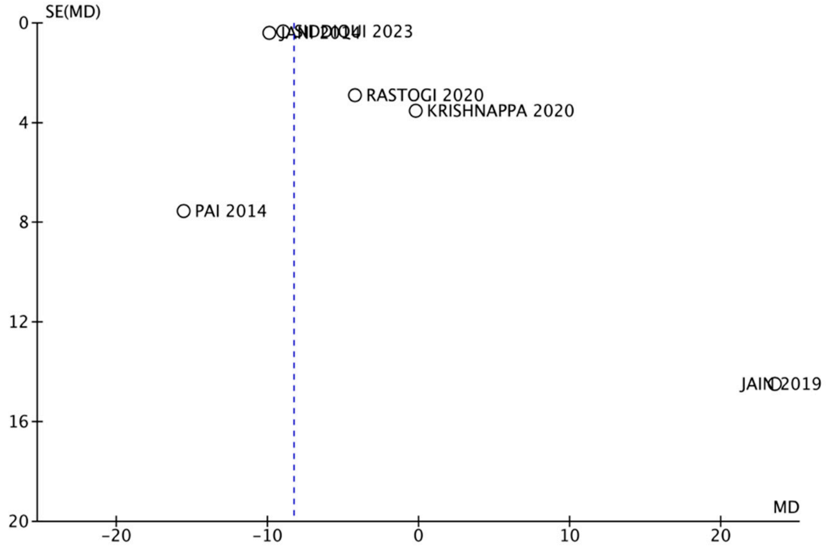

C.

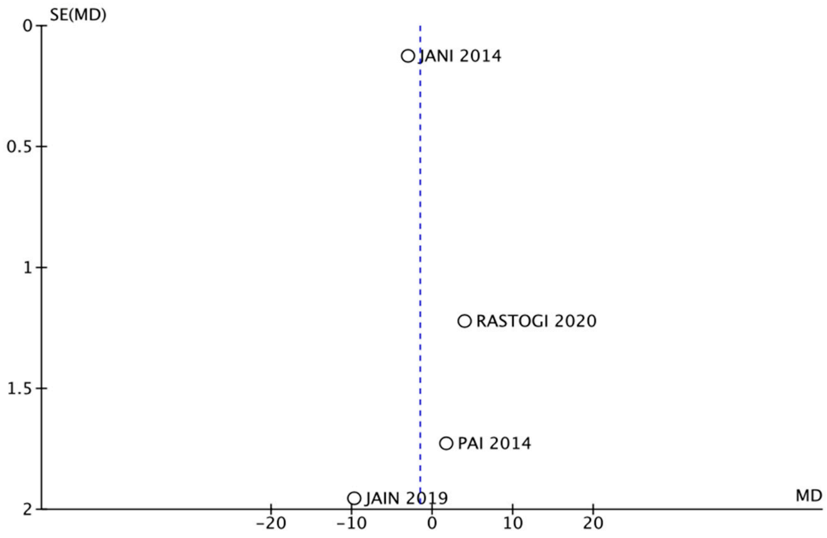
SE(MD)
0
0.5
1
1.5
2
JANI 2014
RASTOGI 2020
PAI 2014
JAIN 2019
MD
−20
−10
0
10
20

D

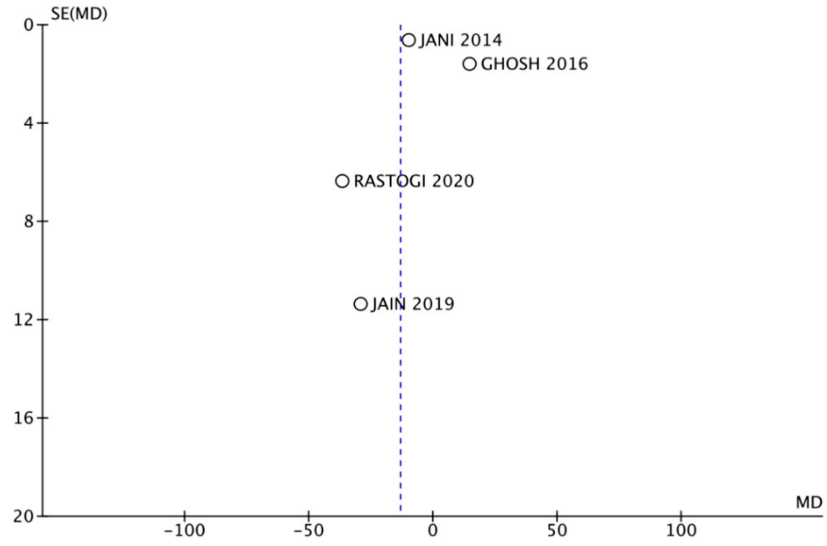
SE(MD)
0
4
8
12
16
20
JANI 2014
GHOSH 2016
RASTOGI 2020
JAIN 2019
MD
−100
−50
0
50
100

Figure S2.
Funnel plots comparing the (A) creatinine level, (B) alanine transaminase level, and (C) body weight between the 2 mg and 4 mg saroglitazar groups. MD: Mean difference; MD: Standard mean difference; SE: Standard error.

A.

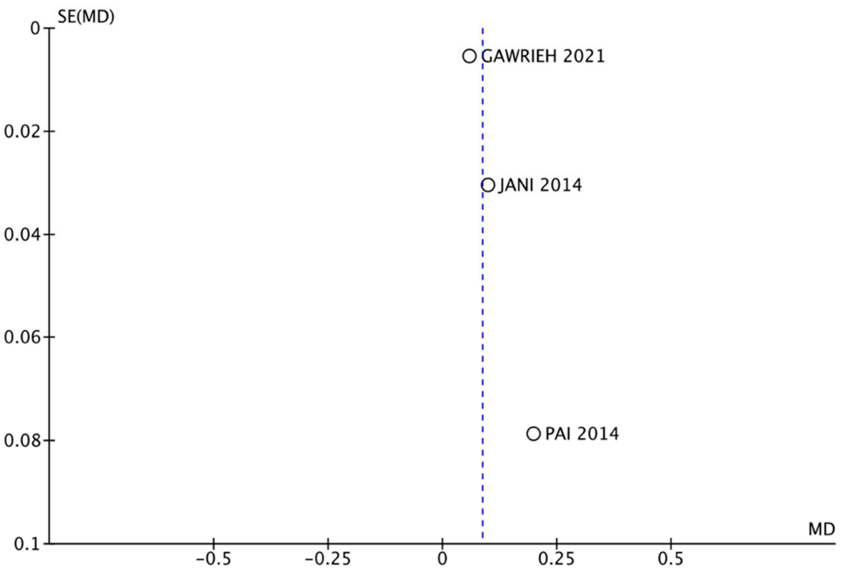

B.

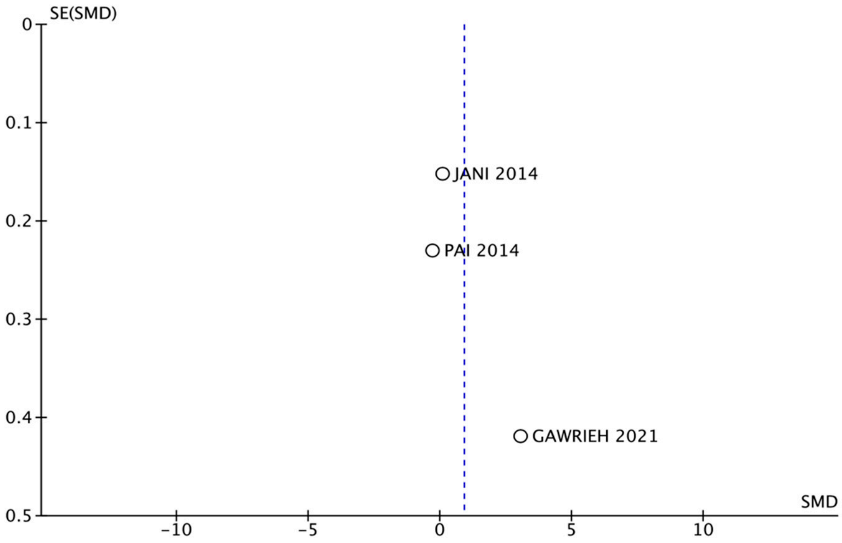

C.

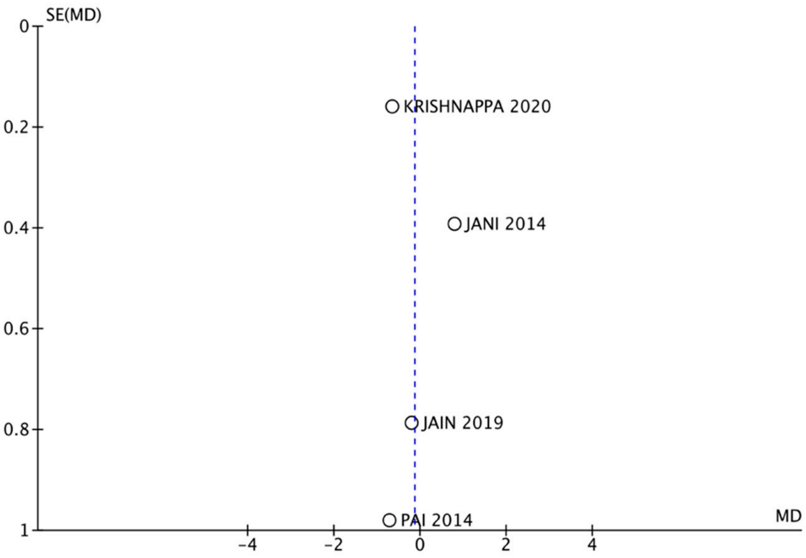
SE(MD)
0
0.2
0.4
0.6
0.8
1
KRISHNAPPA 2020
JANI 2014
JAIN 2019
PAI 2014
MD
-4
-2
0
2
4

Figure S3.

Forest plots comparing efficacy endpoints between the 2 mg and 4 mg saroglitazar groups. A 4-mg dose of saroglitazar (A) significantly decreased the triglyceride level (mg/dL) but did not affect the (B) high-density lipoprotein cholesterol (mg/dL) or (C) low-density lipoprotein cholesterol (mg/dL) levels compared to that in the 2-mg dose group. CI: Confidence interval; IV: Inverse variance; SD: Standard deviation; HDL-C: High density lipoprotein cholesterol; LDL-C: Low density lipoprotein cholesterol.

A. Triglyceride

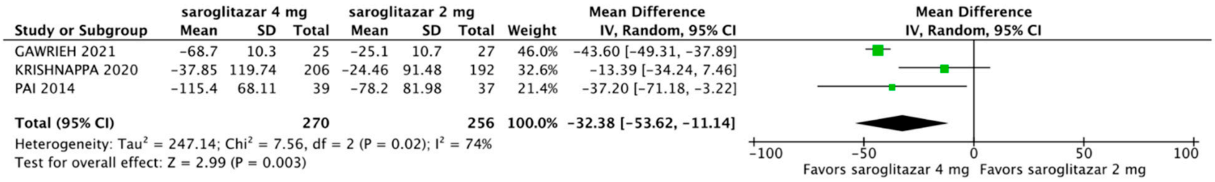

B. HDL-C

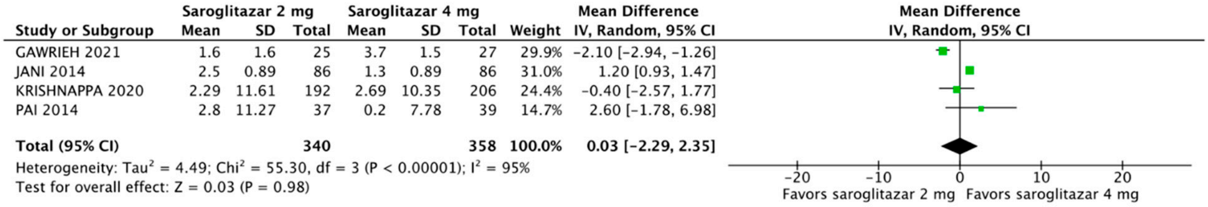

C. LDL-C

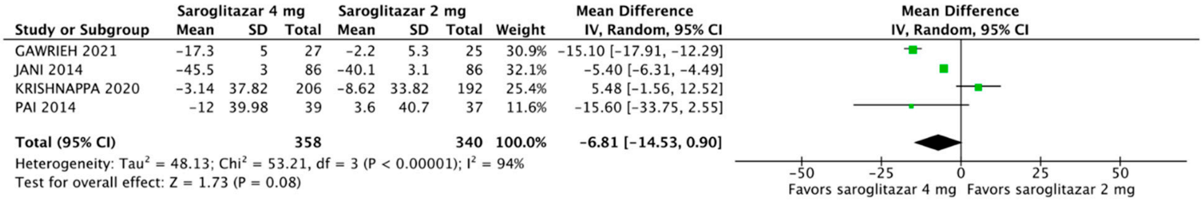

Figure S4.

Forest plot comparing changes in total cholesterol (TC) levels (mg/dL) between saroglitazar 4 mg and control groups. A 4-mg dose of saroglitazar significantly decreased TC.

CI: Confidence interval; IV: Inverse variance; SD: Standard deviation.

Total cholesterol

	Saroglitazar 4 mg			Control				Mean Difference
**Study or Subgroup**	**Mean**	**SD**	**Total**	**Mean**	**SD**	**Total**	**Weight**	**IV, Random, 95% CI**
JAIN 2019	−13.4	32.9	15	−28.4	44.3	15	1.1%	15.00 [−12.92, 42.92]
JANI 2014	−56.4	3.53	86	−40.3	3.38	94	35.5%	−16.10 [−17.11, −15.09]
KRISHNAPPA 2020	−6	40.71	206	−0.47	37.55	206	11.1%	−5.53 [−13.09, 2.03]
PAI 2014	−18.5	40.62	39	9.1	28.77	33	3.2%	−27.60 [−43.69, −11.51]
RASTOGI 2020	−3.6	7.2	10	−0.9	7.8	9	12.9%	−2.70 [−9.47, 4.07]
SIDDIQUI 2023	−22.2	2.7	130	−4.8	3.3	91	36.0%	−17.40 [−18.22, −16.58]
**Total (95% CI)**			**486**			**448**	**100.0%**	**−13.68 [−16.69, −10.66]**

Heterogeneity: $Tau^2 = 6.41$; $Chi^2 = 35.64$, $df = 5$ ($P < 0.00001$); $I^2 = 86\%$

Test for overall effect: $Z = 8.88$ ($P < 0.00001$)

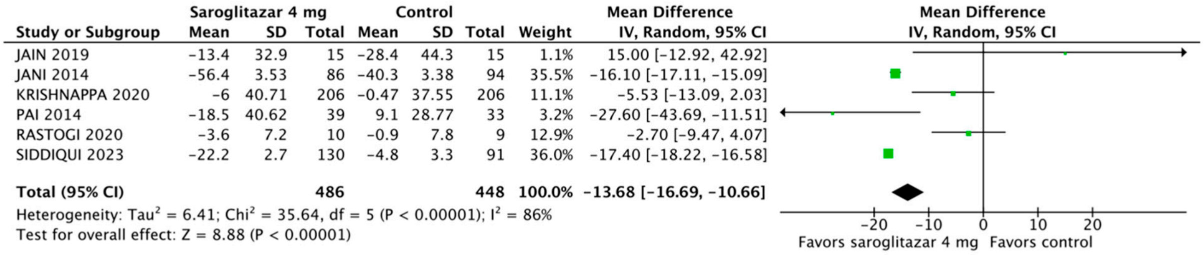

Figure S5.

Forest plots comparing safety endpoints between the 2 mg and 4 mg saroglitazar groups. A 4-mg dose of saroglitazar did not affect the (A) serum creatine level (mg/dL), (B) alanine transaminase level (U/L), or (C) body weight (Kg) compared to that in the 2-mg dose group. CI: Confidence interval; IV: Inverse variance; SD: Standard deviation; ALT (Alanine transaminase)

A. Serum creatinine

	saroglitazar 2mg			saroglitazar 4mg				Mean Difference
**Study or Subgroup**	**Mean**	**SD**	**Total**	**Mean**	**SD**	**Total**	**Weight**	**IV, Random, 95% CI**
JANI 2014	0	0.18	86	0.1	0.2	86	56.4%	−0.10 [−0.16, −0.04]
PAI 2014	0.7	0.21	37	0.7	0.19	39	43.6%	0.00 [−0.09, 0.09]
**Total (95% CI)**			**123**			**125**	**100.0%**	**−0.06 [−0.15, 0.04]**

Heterogeneity: Tau² = 0.00; Chi² = 3.38, df = 1 (P = 0.07); I² = 70%
Test for overall effect: Z = 1.14 (P = 0.26)

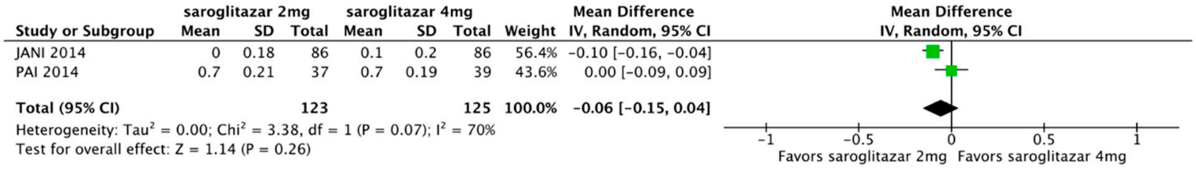

B. ALT

	saroglitazar 4 mg			saroglitazar 2 mg				Std. Mean Difference
**Study or Subgroup**	**Mean**	**SD**	**Total**	**Mean**	**SD**	**Total**	**Weight**	**IV, Random, 95% CI**
GAWRIEH 2021	−27.7	5.9	25	−45.8	5.7	27	31.4%	3.08 [2.25, 3.90]
JANI 2014	1.4	57.8	86	−4.3	33.3	86	34.6%	0.12 [−0.18, 0.42]
PAI 2014	−0.2	0.3	39	−0.1	0.36	37	34.0%	−0.30 [−0.75, 0.15]
**Total (95% CI)**			**150**			**150**	**100.0%**	**0.91 [−0.50, 2.31]**

Heterogeneity: Tau² = 1.45; Chi² = 51.89, df = 2 (P < 0.00001); I² = 96%
Test for overall effect: Z = 1.27 (P = 0.21)

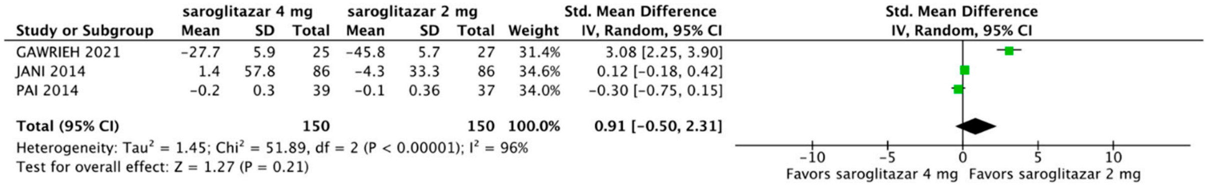

C. Weight

	Saroglitazar 4 mg			Saroglitazar 2 mg				Mean Difference
**Study or Subgroup**	**Mean**	**SD**	**Total**	**Mean**	**SD**	**Total**	**Weight**	**IV, Random, 95% CI**
JANI 2014	0.3	2.83	86	−0.6	2.63	86	35.5%	0.90 [0.08, 1.72]
KRISHNAPPA 2020	−0.05	1.67	206	0.07	1.5	192	51.0%	−0.12 [−0.43, 0.19]
PAI 2014	−0.1	2.7	39	−0.8	5.35	37	13.6%	0.70 [−1.22, 2.62]
**Total (95% CI)**			**331**			**315**	**100.0%**	**0.35 [−0.46, 1.17]**

Heterogeneity: Tau² = 0.31; Chi² = 5.72, df = 2 (P = 0.06); I² = 65%
Test for overall effect: Z = 0.85 (P = 0.40)

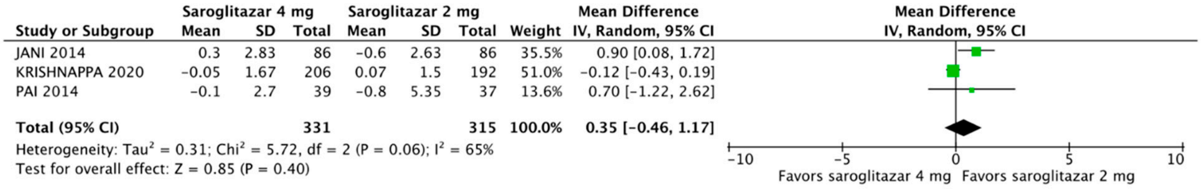

Supplement: Supplementary file 1 [file jcm-12-05674-s001.zip › jcm-2585258-supplementary.pdf]
